# Pancreatic mucinous cystadenoma with serum CA 19–9 *over* 1,000,000 U/mL: a case report and review of the literature

**DOI:** 10.1186/s12957-015-0476-y

**Published:** 2015-02-25

**Authors:** Wilson L Costa, Henrique Mantoan, Rafael Horácio Brito, Héber SC Ribeiro, Alessandro L Diniz, André Luís Godoy, Igor Correia Farias, Maria Dirlei FS Begnami, Fernando Augusto Soares, Felipe JF Coimbra

**Affiliations:** Department of Abdominal Surgery, A. C. Camargo Cancer Center, Rua Antonio Prudente 211, Liberdade CEP 01501-900 São Paulo, Brazil; Department of Surgical Pathology, A. C. Camargo Cancer Center, São Paulo, Brazil

**Keywords:** Pancreatic cystic lesions, Biomarkers, CA19-9

## Abstract

**Background:**

The diagnosis of pancreatic cystic neoplasms has become more accurate recently. In some cases, however, doubt remains regarding the lesion’s malignant potential. CA 19–9 has long been identified as a reliable biomarker in differentiating pancreatic benign and malignant lesions, especially in non-jaundiced patients.

**Case report and discussion:**

We report a case of a young female who presented with a mucinous lesion in the tail of the pancreas and a serum CA 19–9 over 1,000,000 U/mL. She was taken to surgery and had a distal pancreatectomy and splenectomy. Pathology reports showed only a mucinous cystadenoma. After 1 year of follow-up, her serum CA 19–9 was normal. Following that, the work-up in these lesions, the role of the biomarker in pancreatic adenocarcinoma and in the differentiation between benign and malignant lesions is discussed.

## Background

Pancreatic cystic neoplasms (PCNs) still constitute a challenge in everyday clinical practice. Current imaging diagnostic methods, increasingly more sophisticated and associated with an ever-increasing spectrum of serum biomarkers or cystic content analysis, are still not able to differentiate reliably enough the benign lesions, amenable to follow-up, from those malignant or premalignant lesions that require surgical treatment.

## Case presentation

**Figure 1:**
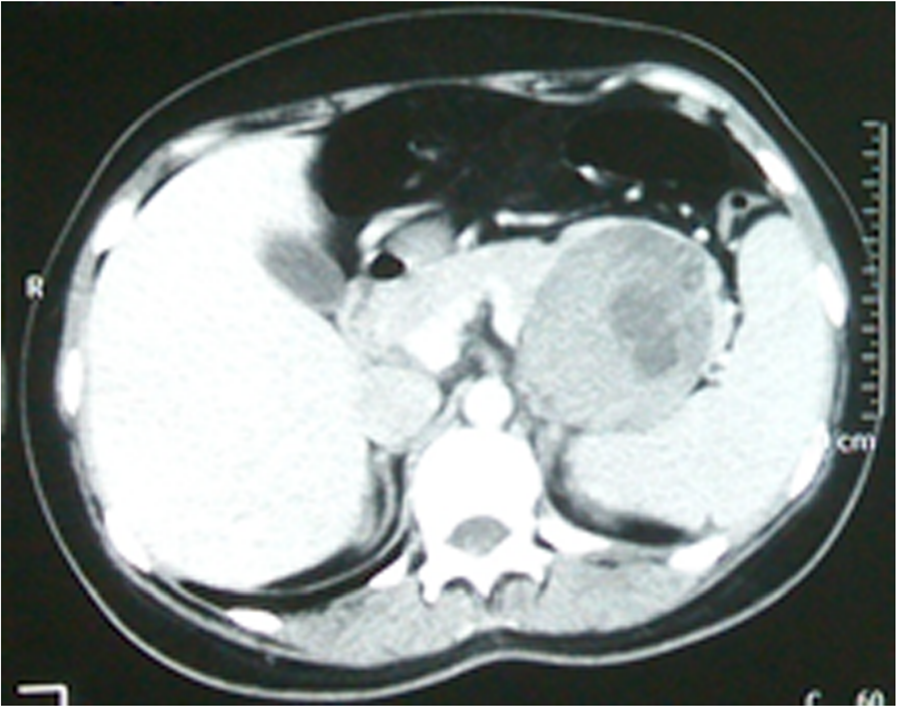
**Abdominal CT with IV contrast showing neoplasm of the pancreatic tail in close contact with the spleen.**

A female patient, 39 years old, is complaining of diffuse abdominal pain, more prominent in the flank and left hypochondrium, initially bearable and with response to simple analgesics, but with progressive and significant worsening in the last 24 h. The patient reported bilious vomiting and denied fever or other similar episodes in the past. She was admitted in good general condition, moderately dehydrated and discolored. On abdominal palpation, there was evidence of diffuse pain without peritonitis. The patient was given IV hydration with crystalloids and underwent an abdominal/pelvic CT scan, which identified a voluminous expansive formation in the pancreatic tail, with compression, but without direct invasion of the splenic vein (Figure 1). Laboratory tests showed CA 19–9 serum concentration greater than 1,000,000 U/mL, with no other changes.

Upper gastrointestinal (UGI) endoscopy did not show any extrinsic compression or gastric/duodenal wall invasion.

**Figure 2:**
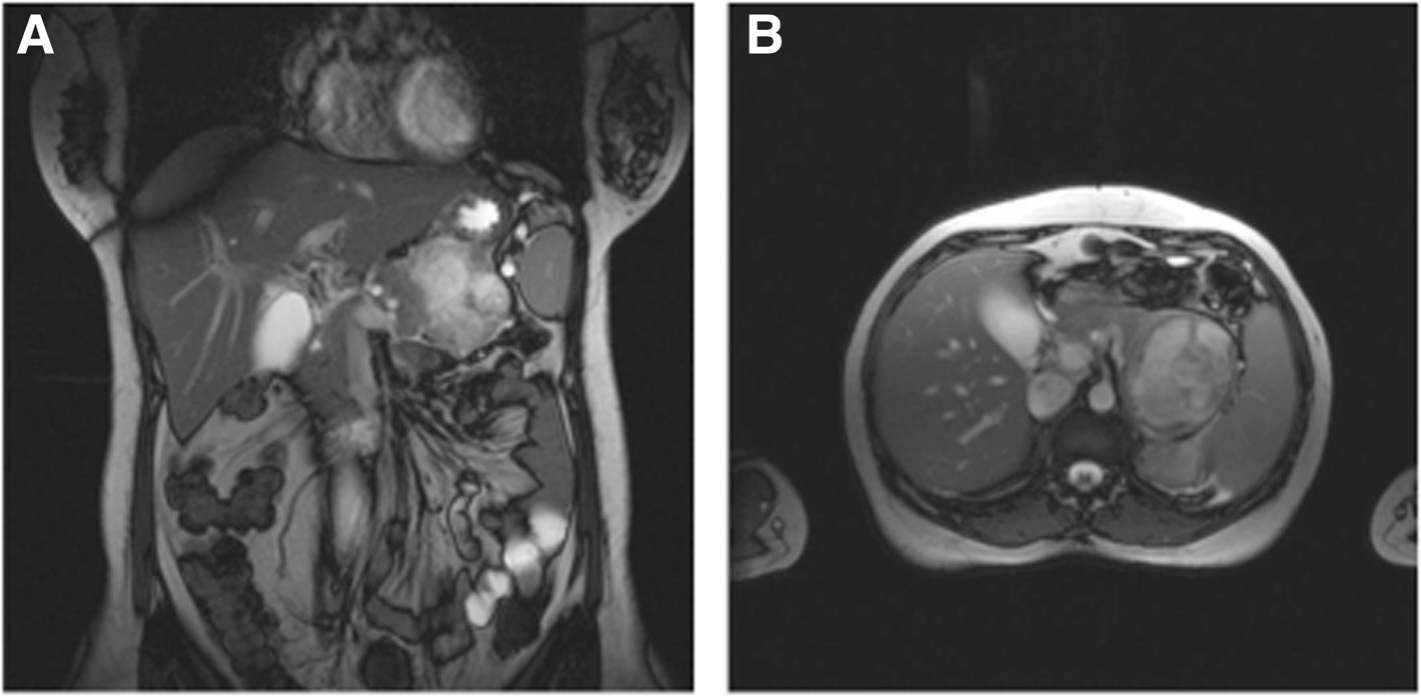
**MRI of the upper abdomen showing the lesion in the pancreatic tail. (A)** View in coronal plane. **(B)** View in axial plane.

An MRI of the upper abdomen was performed in order to better characterize the lesion, with evidence from projection imaging of a mass in the pancreatic tail, predominantly with cystic aspect, multiloculated, with thick septations and regular margins, showing heterogeneous contrast enhancement and areas of T1 signal hyperintensity, possibly corresponding to hematic content. Its size was 63 × 60 × 61 mm (Figure 2).

No endoscopic ultrasound was indicated, since the main diagnostic hypothesis was a mucinous lesion with malignant transformation, and its treatment included surgery, provided that no metastatic disease was found.

Clinical stabilization and preoperative evaluation was performed, with no additional significant findings. The patient was the taken to the operating room. At first, she underwent a diagnostic laparoscopy, with no signs of peritoneal and liver metastasis. Laparotomy was then performed, and a distal pancreatectomy with splenectomy was conducted. The patient had an uneventful postoperative outcome and was discharged on the fifth postoperative day.

The final pathology report provided the diagnosis of a mucinous cystic neoplasm with moderate dysplasia and no signs of invasion.

Its macroscopic exam described a 6-cm well-delineated cystic tumor surrounded by a thick, fibrotic capsule. In its composition, multiple cysts with trabecular and thickened septa were observed. The cysts contents were mucoid and showed no areas of solid tumor. Degenerative changes, including hemorrhage and macrocystic degeneration were seen in focal areas.

**Figure 3:**
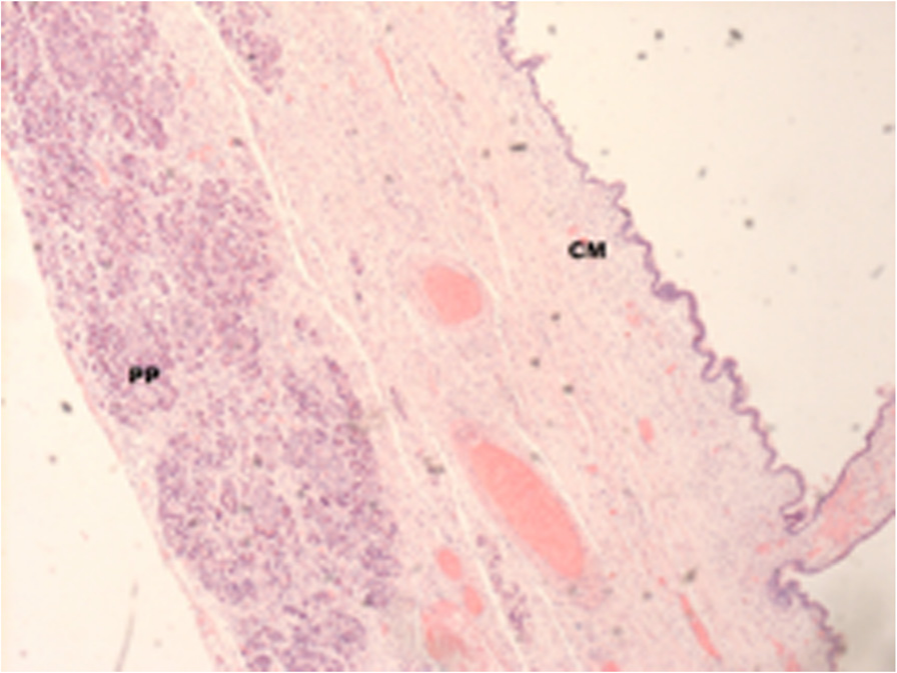
**Hematoxylin-eosin staining,** **×** **100 magnification.** View showing the relationship of normal pancreatic parenchyma (PP) with mucinous cystadenoma (CM).

**Figure 4:**
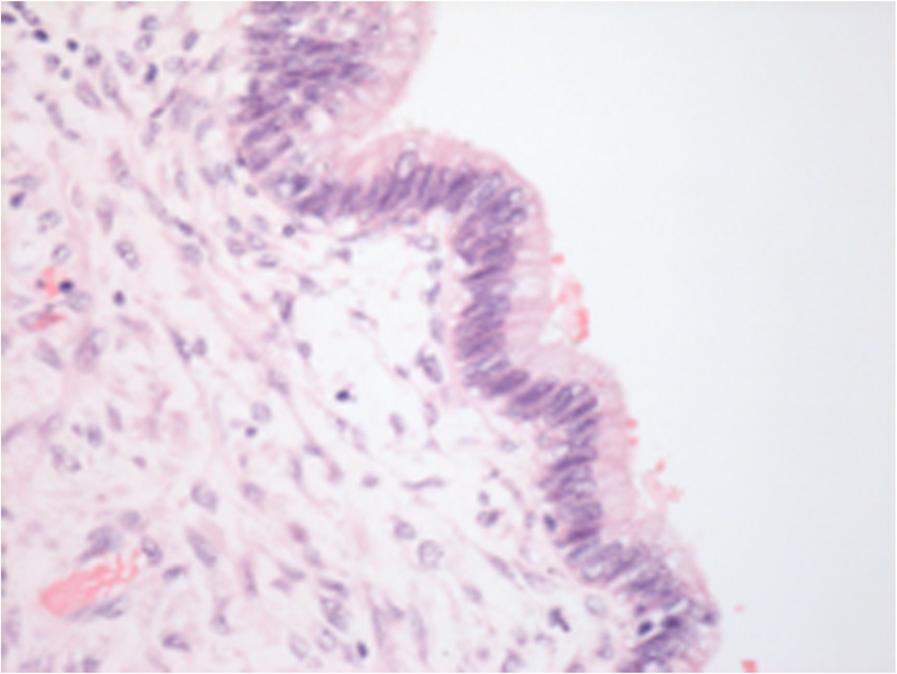
**Hematoxylin-eosin staining, ×1,000 magnification.** Detail of the dysplastic area of the cyst epithelium is shown.

Microscopically, the epithelial lining was consisted of tall, columnar cells with frequent apical mucin. The tumor was mostly largely bland in appearance, containing uniform, basally oriented nuclei. Focally, it exhibited architectural complexity with pseudostratified hyperchromatic nuclei, but no invasive carcinoma was found. The subepithelial stroma was hypercellular containing spindle cells resembling the stroma of the ovary (ovarian-like stroma) (Figures 3 and 4).

On the 40th postoperative day, the patient had an outpatient visit, reporting no symptoms and without any change in blood glucose levels. The CA 19–9 serum concentration at that time was 688 U/mL. Additionally, the patient brought a complete abdominal ultrasonography showing only postoperative changes.

After 5 months, the patient remained without complaints and her CA 19–9 serum concentration was still above normal (56 U/mL).

In a late postoperative visit, 1 year after surgery, her CA 19–9 serum concentration had returned to normal levels (35.2 U/mL). No abnormal events have been recorded since, with 3 years of follow-up.

## Discussion

A controversy has been observed regarding the appropriate indication for surgery in some pancreatic tumors, with some authors identifying up to 30% of unnecessary resections [1]. Differentiation of pancreatic tumors between benign and malignant, whether cystic, solid, or mixed lesions, is essential for the best course of treatment. PCNs remain a perfect example of tumors that present in a variable way and in which surgical treatment should be tailored. Their incidence has been increasing through the years, albeit with a proportional reduction in the finding of malignant lesions [2]. Therefore, the knowledge of the varieties of PCNs, as well as the correct differentiation between inflammatory or serous lesions, which present no potential for malignant transformation and those of mucinous lesions which are premalignant [3], is mandatory for choosing the appropriate therapeutic approach.

The widespread use of imaging tools in differentiating these tumors has provided safe diagnosis for typical lesions; however, many cystic tumors show intermediate characteristics between benign and malignant ones. Even with the use of modern methods of diagnostic imaging, such as contrast-enhanced ultrasonography and endoscopic ultrasonography associated with fine needle aspiration (FNA), which are also important in this differentiation [4,5], the decision between expectant and surgical course of treatment will also involve factors beyond imaging characteristics, such as serum biomarker concentrations, patient status, and the presence or absence of symptoms [6,7].

An ‘ideal’ tumor biomarker is highly sensitive in identifying disease in an asymptomatic population. CA 19–9, a serum carbohydrate antigen produced by exocrine epithelial cells, was first described more than three decades ago [8]. It is the only blood test approved by the Food and Drug Administration (FDA) for pancreatic adenocarcinoma. CA 19–9 has demonstrated specificity of approximately 85% and sensitivity of 90% [9,10], and its prognostic value for survival is more associated with high postoperative dosage [11].

In evaluating benign and malignant intraductal papillary mucinous neoplasms (IPMNs), CA 19–9 has a role in the differentiation invasive and benign lesions. In a study with 142 patients, 80% of those with invasive IPMNs had CA 19–9 over 37 U/ml versus only 18% of those with benign lesions. The specificity was 85.9% and accuracy 81.7% [12]. A cancer center series of 287 resected patients with the diagnosis of IPMN reported preoperative carcinoembryonic antigen (CEA) and CA 19–9 serum concentrations in 112 patients. Among them, one biomarker was elevated in 23 individuals (20.5%), of which 16 (69%) had invasive tumor in the final anatomopathological result (*P* < 0.01). The authors advocated that Sendai criteria-negative patients who present elevated CA 19–9 be considered for surgery [13].

Two other common cystic neoplasms, serous and mucinous cystoadenomas, have a different pattern of CA 19–9 concentrations. In an American study with 114 patients, CA 19–9 was rarely elevated in serous lesions, but was associated with increases of up to sixfold in mucinous ones, with the presence of symptoms being the most sensitive criterion for malignancy. The cut-off value used was 35 mg/dL [14].

In patients with resectable ductal adenocarcinoma, the increase of CA 19–9 has had an impact on staging and survival. In a large series of 424 patients, tumors that were larger and had lymph node metastasis were associated with a median CA 19–9 over 160 U/mL. Also, individuals with CA 19–9 over 1,000 U/mL had median survival time of only 1 year [15]. Similar findings were observed in a British series with 109 subjects, but a different cut-off value was identified. Patients with a CA 19–9 level over 150 kU/L had worse overall survival, poorly differentiated tumors, and more commonly had a resection with positive margins [16]. For patients undergoing neoadjuvant chemotherapy prior to resection, it was demonstrated that those with serum concentrations of CA 19–9 greater than 1,167 U/mL had a survival rate around 8 months [17].

It must be reminded that some patients, while having invasive neoplasm, do not have an increase in CA 19–9, as 5% to 7% of the population has the Lewis blood group phenotype Le(a-b-) that does not synthesize the antigen detected by the antibody CA 19–9 [18].

Further tools have been investigated in order to earlier detect cysts that tend to progress. Genetic analysis of their content constitutes a horizon of particular interest. The concentrations of the CEA biomarker in the cyst can help differentiate between serous and mucinous lesions, but cannot, however, infer malignancy [19]. Detection of *K-RAS* gene mutation and loss of amplitude in allele expression in cyst fluid confer specificity up to 96% for the diagnosis of mucinous malignant lesions, but with specificity as low as 37% [4].

The PAM4 marker, an antigen produced by tumor cells, shows a sensitivity of 76% with a specificity of 96% in the diagnosis of pancreatic adenocarcinoma, including in the early stages of onset. The association of CA 19–9 concentrations to PAM4 conferred 84% specificity in diagnosis, regardless of serum bilirubin concentrations [20].

One scenario in which CA 19–9 results must be interpreted with caution is in jaundiced patients. Those who present with benign pathologies of the biliopancreatic tract coursing with jaundice have CA 19–9 concentrations above 37 U/mL at a frequency of up to 64.7% versus 7.3% in non-jaundiced patients, showing the limited potential of CA 19–9 concentrations in the differential diagnosis between benign and malignant conditions in jaundiced individuals [21].

In the case in question, no focal point of invasion was identified and the patient remained anicteric throughout the course of the disease. The presence or absence of dysplasia, and the degree thereof, has shown no correlation with the serum concentrations of biomarkers. This just confirms the authors’ findings that in spite of the vast evolution of diagnostic methods, whether originating from imaging or the laboratory, the correlation between the preoperative diagnosis and the postoperative histological diagnosis of the surgical specimen still revolves around 78.4% [22].

## Conclusions

In the current report, a case is presented in which a young female patient has a single pancreatic cystic lesion in the pancreatic tail and also preoperative serum marker of 1,000,000 U/ml, whose pathology report identified just a mucinous cystoadenoma, with no invasive tumor. This further demonstrates that biomarkers should be used as a diagnostic and staging aid and should not be used for screening and/or defining treatment course.

## Consent

Written informed consent was obtained from the patient for publication of this case report and any accompanying images. A copy of the written consent is available for review by the Editor-in-Chief of this journal.
